# Genetic or familiar forms of primary hyperparathyroidism: description of a case series with familial isolated hyperparathyroidism and review of the literature

**DOI:** 10.20945/2359-4292-2024-0311

**Published:** 2025-08-18

**Authors:** Miguel Madeira, Maria Caroline Alves Coelho, Leandro Kasuki, Filipe Barbosa Linhares, Isabel Sampaio Tostes, Rafael Mazzutti Dutra Santana, Raquel Beatriz Gonçalves Muniz, Maria Lucia Fleiuss de Farias, Fernanda Vaisman

**Affiliations:** 1 Programa de Pós-graduação em Endocrinologia, Departamento de Clínica Médica, Hospital Universitário Clementino Fraga Filho/Universidade Federal do Rio de Janeiro, Rio de Janeiro, RJ, Brasil; 2 Divisão de Endocrinologia, Departamento de Clínica Médica, Hospital Federal de Bonsucesso, Rio de Janeiro, RJ, Brasil; 3 Programa de Pós-graduação em Fisiopatologia Clínica e Experimental (FISCLINEX), Universidade do Estado do Rio de Janeiro, Rio de Janeiro, RJ, Brasil; 4 Divisão de Endocrinologia, Departamento de Clínica Médica, Faculdade de Ciências Médicas, Universidade do Estado do Rio de Janeiro, Rio de Janeiro, RJ, Brasil; 5 Unidade de Neuroendocrinologia, Instituto Estadual do Cérebro Paulo Niemeyer, Rio de Janeiro, RJ, Brasil; 6 Divisão de Endocrinologia, Instituto Nacional de Câncer José Alencar Gomes da Silva (INCA), Rio de Janeiro, RJ, Brasil

**Keywords:** Primary hyperparathyroidism, parathyroid neoplasms, parathyroid cancer, CDC73 protein

## Abstract

Primaryhyperparathyroidism (PHPT) is a disorder of mineral metabolism caused by inappropriate or excessive secretion of parathyroid hormone. It occurs sporadically in approximately 95% of cases but may also be associated with complex syndromes and/or a familial (i.e., hereditary) history. We report the clinical, laboratory, and genetic profiles of a case series with familial isolated hyperparathyroidism. Diagnosis was established in patients aged 22-41 years (median = 32), and recurrence was identified in four patients (three with adenoma and one with hyperplasia and parathyroid carcinoma). Six family members presented with a heterozygous mutation in the CDC73 gene, and one patient had a copy number variation of undetermined clinical significance in the same gene. In addition, we review the particularities of each condition associated with PHPT, indications for genetic evaluation, and recommendations for follow-up and treatment.

## INTRODUCTION

Primary hyperparathyroidism (PHPT) is characterized by elevated or inappropriately normal levels of parathyroid hormone (PTH) associated with increased serum calcium concentrations. This disease results from excessive production of PTH by one or more parathyroid glands, leading primarily to various complications in bones and kidneys (^1^). The majority of PHPT cases are sporadic, with fewer than 5% having a family history. These familial cases are generally associated with germline mutations in genes known to confer susceptibility to parathyroid tumor development (^2^).

In hereditary or familial forms, PHPT typically presents as part of a syndrome, the most common being multiple endocrine neoplasia type 1 (MEN1) (^3^). PHPT also occurs in multiple endocrine neoplasia type 2 (MEN2), multiple endocrine neoplasia type 4, hyperparathyroidism-jaw tumor syndrome, familial hypocalciuric hypercalcemia (FHH), and familial isolated hyperparathyroidism (FIHP).

FIHP is a rare hereditary disorder with an autosomal dominant inheritance pattern, characterized by PHPT in the absence of other diseases or tumors, such as those observed in the aforementioned syndromes. Accurate identification of a family with FIHP is crucial for appropriate follow-up, with significant implications for the treatment and genetic counseling of patients and their relatives (^4^).

In this study, we report a family comprising 12 individuals, including several cases of PHPT unaccompanied by other associated conditions. Along with clinical and laboratory assessments, we conducted a genetic evaluation of the major genes implicated in familial hyperparathyroidism syndromes and compared our findings with the existing scientific literature.

## SUBJECTS AND METHODS

Our study examined 12 members of a three-generation family with cases of PHPT. During the initial evaluation and subsequent follow-up, comprehensive clinical surveillance, as well as laboratory and imaging tests, were performed, confirming the absence of evidence for other genetic syndromes related to familial PHPT. All patients diagnosed with PHPT were evaluated for calcium metabolism (including 24-hour urinary calcium excretion), pituitary hormonal abnormalities, and underwent abdominal and pelvic tomography, as well as cervical ultrasound.

### DNA extraction

Blood samples were collected in the morning after an 8-hour fast. DNA extraction was performed within 72 hours of blood collection. Genomic DNA was extracted from 300 µL of whole blood using the PureGene Blood Kit (Gentra, Minneapolis, MN), according to the manufacturer’s instructions, and reconstituted in 100 µL of DNA Hydration Solution provided with the kit.

### Genetic analysis

Genetic analysis was conducted using next-generation sequencing to assess the following genes: CASR (calcium-sensing receptor), CDC73 (cell division cycle 73), CDKN1B (cyclin-dependent kinase inhibitor 1B), GCM2 (glial cells missing transcription factor 2), MEN1 (menin 1), and RET (ret proto-oncogene). **Table 1** summarizes the evaluated genes and their main characteristics.

**Table 1:** Genes evaluated, main characteristics and associated syndromes

Gene	Gene location	Mechanism	Syndrome
MEN1	11q13	TS, LoF	MEN1
RET	10q11.21	PO, GoF	MEN2A
CDKN1B	12p13.1	TS, LoF	MEN4
CDC73	1q31.2	TS, LoF	HPT-JT, FIHP
CaSR	3q13.3-q21.1	ICSST, LoF	FHH, FIHP
GCM2	6p24.2	PO, GoF	FIHP

Adapted from Reference 2. TS: tumor suppressor gene; LoF: loss of function; MEN: multiple endocrine neoplasia; PO, proto-oncogene; GoF, gain of function; HPT-JT: hyperparathyroidism jaw tumor syndrome; FIHP: familial isolated hyperparathyroidism; ICSST: impaired calcium sensing or signal transduction FHH: familial hypocalciuric hypercalcemia.

## RESULTS

Among the 12 patients who underwent genetic research, we included the asymptomatic spouse of the index case. The spouse was screened; however, they did not present any symptoms nor had a diagnosis of PHPT, demonstrating normal examination results and did not have mutations related to PHPT. Among the other ten family members, seven were asymptomatic, and the analyses were performed as part of a cascade screening.

**Figure 1:**
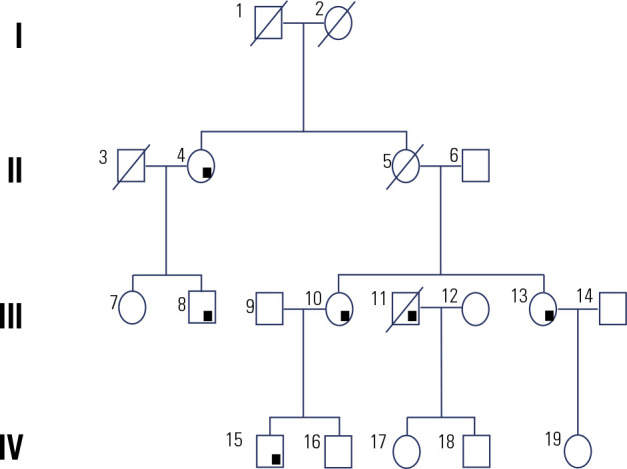
Family pedigree.

The diagnosis of PHPT was established between the ages of 22-41 years (median = 32 years), and recurrence was identified in four patients. **Figure 1** demonstrates the family pedigree, and **Table 2** presents the clinical and laboratory characteristics of all patients with CDC73 mutations. Below, we describe the clinical history of patients with various manifestations of PHPT.

**Table 2:** Clinical and laboratory characteristics of all family members with the mutation in the CDC73 gene

No.	Sex	Age at diagnosis (years)	Initial presentation	Preoperative PTH^*^	SerumCa pre-op^**^	PH	Outcome
II.4	F	30	Asthenia, femoral lame pain	184	14.9	Adenoma	Recurrence after 11 years > second surgery. Hypercalciuria in treatment.
III.8	M	28	Asthenia	279	12.42	Adenoma	Recurrence after 13 years with renal hypercalciuria
III.10	F	41	Asymptomatic	118	10.5	Adenoma	In remission for 17 years
III.11	M	32	Asthenia and nephrolithiasis	310	12.8	Hyperplasia and carcinoma	Recurrence with parathyroid carcinoma and metastasis; died due to complications from the disease
III.13	F	40	Asymptomatic	234	12.9	Adenoma	Recurrence after 12 years > second surgery > in remission for 7 years
IV.15	M	22	Asymptomatic	83	9.6	Adenoma	In remission for 14 years

F: Female; M: male. PH: parathyroid histology.

* Reference value = 7-53 pg/mL.

** Reference value = 8.8-10.2 mg/dL.

Square: males; circle: females; diagonal slash: deceased; black square: heterozygous mutation in the CDC73 gene.

A heterozygous pathogenic variant was found in the CDC73 gene, characterized by the replacement of aspartate with tyrosine at codon 90 in seven family members. This mutation was initially described as a clinical variant of undetermined significance. Since these patients had a confirmed diagnosis of PHPT, we can consider that this mutation, previously described as a variant of undetermined significance, is in fact pathogenic. Copy number variations of undetermined clinical significance were identified in one family member (19 years old, patient IV.18), who remained asymptomatic (no clinical or laboratory evidence of PHPT) at the time of evaluation.

### III.11 (index case)

A 32-year-old male patient was diagnosed with PHPT in 1999 due to joint pain, fatigue, nausea, and nephrolithiasis, which led to subsequent left nephrectomy. In 2002, he underwent a partial parathyroidectomy, with removal of three parathyroid glands and half of the fourth, as well as autotransplantation in the left upper limb. Histopathology (HP) revealed parathyroid hyperplasia. He experienced relapses with subsequent parathyroid surgeries in 2004, 2006, and 2010, with only hyperplasia identified in the HP from these procedures.

In February 2011, the patient was hospitalized with body aches, nausea, and headache, associated with hypercalcemia. Initially, clinical treatment was conducted to control hypercalcemia, and the fifth surgery was performed in May 2011, at which time metastatic parathyroid carcinoma was diagnosed. Histopathology identified a 3.7 cm parathyroid tumor, a 2.5 cm metastatic paratracheal lesion with vascular invasion, compromised soft tissue surgical margins, and metastasis in a cervical lymph node. The patient also underwent postoperative radiotherapy and treatment for hungry bone syndrome. He experienced persistent hypocalcemia until October 2011, when PTH and calcium levels increased again. A chest computed tomography scan revealed a cystic lesion in the posterior segment of the upper lobe of the left lung.

Clinical control of calcium was initially managed with cinacalcet, furosemide, and zoledronic acid; however, due to progressive increases in PTH and calcium, a new chest computed tomography scan was performed, which demonstrated the appearance of new thoracic lesions. Four additional surgical interventions were performed to excise the metastases, and the patient was monitored clinically. He subsequently died due to complications related to hypercalcemia and progression of tumor lesions.

### II.4

A female patient presented with weakness, adynamia, and pain in both hip joints, with restricted walking, beginning at 30 years of age. During her first consultation, a palpable nodule was detected in the lower aspect of the thyroid region. A bone biopsy provided histopathological evidence of fibrous cystic osteitis, leading to the diagnosis of PHPT. She experienced a spontaneous fracture of the left femur during hospitalization for surgical treatment of PHPT. Thereafter, a right lower parathyroidectomy was performed, with identification of a parathyroid adenoma. During surgery, the superior parathyroid glands were not identified.

In the postoperative period, she experienced transient hypoparathyroidism and lost outpatient follow-up, returning only 11 years after the first surgery. At that time, she presented with hypercalcemia and a left superior nodule identified by cervical ultrasound. Surgical treatment was indicated, and the upper left parathyroid was removed (HP diagnosis of parathyroid adenoma). She continued to have renal hypercalciuria, necessitating initiation of hydrochlorothiazide therapy. She is currently monitored for osteopenia (diagnosed thirty years after the first surgery), with normal PTH and calcium levels.

### III.8

A 28-year-old male patient presented with complaints of asthenia and cramps, accompanied by laboratory tests PHPT. Scintigraphy and ultrasound suggested the presence of a parathyroid adenoma in the lower left parathyroid gland. One year later, a left parathyroidectomy was performed, along with a partial thyroidectomy on the same side. The diagnosis of parathyroid adenoma was confirmed, and normal thyroid tissue was observed. He remains asymptomatic, with normal PTH and calcium levels.

### III.10

A 41-year-old female patient was referred for PHPT screening. She was asymptomatic but had elevated PTH and calcium levels. Scintigraphy results were normal; however, ultrasound revealed bilateral nodules suggestive of parathyroid enlargement. A subtotal thyroidectomy and parathyroidectomy were performed, involving resection of all four parathyroid glands, with implantation of half of the upper left parathyroid into the left sternocleidomastoid muscle. Histopathological examination identified parathyroid adenomas and normal thyroid tissue. The patient experienced transient hypoparathyroidism, after which she remained asymptomatic, with normal PTH and calcium levels.

### III.13

A 40-year-old asymptomatic female patient attended a screening consultation. Physical examination revealed a nodule in the lower third of the thyroid region. Initial laboratory evaluation showed hypercalcemia, and ultrasound suggested enlargement of the right upper and lower parathyroid glands. Surgical intervention included removal of both glands, with histopathology confirming adenomas in both. Twelve years later, she experienced a recurrence of PHPT; scintigraphy was normal, but ultrasound indicated an enlargement of the lower left parathyroid. Surgery was performed to remove the lower left parathyroid gland. The patient remains under clinical follow-up, without any clinical or laboratory evidence of PHPT.

### IV.15

An asymptomatic male patient underwent his first evaluation for PHPT screening at 22 years old. Initial laboratory investigations revealed increased PTH and calcium levels, and cervical ultrasound identified a nodule suggestive of parathyroid enlargement. Scintigraphy further suggested a parathyroid adenoma in the lower left side. The patient missed subsequent appointments and returned two years later, at which time surgical resection of the adenoma was performed. Since then, no clinical or laboratory findings of PHPT have been identified.

## DISCUSSION

This report describes a case series of FIHP in a three-generation Brazilian family comprising 12 members, of whom seven, aged 22-41 years, were diagnosed with FIHP. In six members, a heterozygous mutation in the CDC73 gene was identified, while copy number variations of undetermined clinical significance were observed in one patient. The affected individuals exhibited PHPT without any clinical or biochemical evidence of MEN1, MEN2, FHH, or hyperparathyroidism-jaw tumor syndrome, thereby fulfilling the diagnostic criteria for FIHP. Since FIHP was first described in the 1930s, its evaluation and definition have posed challenges, largely due to the small number of affected relatives and the presence of mildly symptomatic cases. In our study, a greater number of affected relatives was identified than previously reported in the literature, which documents an average of two affected relatives per family (^5^).

Genetic testing plays a key role in identifying mutations associated with inherited syndromes involving PHPT. Such testing aids in distinguishing hereditary from sporadic cases and enables early detection of individuals at risk for other associated diseases within these syndromes, even before the onset of clinical manifestations. Furthermore, genetic tests help confirm the diagnosis in cases where patients with PHPT present with additional diseases or neoplasms, such as pituitary or pancreatic nodules or jaw tumors. Genetic testing is also crucial for identifying family members who carry the same genetic mutation so that they can receive appropriate monitoring and treatment, even in the absence of symptoms. For unaffected family members, a negative genetic test result can eliminate the need for ongoing follow-up or further investigations, thereby reducing both costs and anxiety for these individuals (^2^). **Table 3** presents the indications for genetic testing in PHPT patients.

**Table 3:** Indications for testing for genetic mutations in PHPT patients

No.	Description
1	PHPT occurring before 45 years
2	Multigland disease
3	Parathyroid carcinoma or atypical parathyroid adenomas (e.g., with fibrous bands or cysts)
4	Being a first-degree relative of a known mutation carrier
5	Being an index case with two or more MEN syndrome-associated endocrine tumors

Adapted from Reference 6. PHPT: primary hyperparathyroidism; MEN: multiple endocrine neoplasia.

Our index patient met the first three criteria described above. The identification of a CDC73 pathogenic variant in this patient led to further research involving other family members. CDC73 mutations are found in 5%-10% of probands presenting with FIHP and in 20%-30% of patients with sporadically presenting parathyroid carcinoma (^4^). Some individuals had already been diagnosed with PHPT, while others were asymptomatic or oligosymptomatic at the time of genetic analysis. The CDC73 gene encodes parafibromin, a tumor suppressor protein. CDC73 pathogenic variants are also associated with a higher frequency of parathyroid carcinoma (^4^), as observed in our index case.

In genetic or familial forms, PHPT typically arises as part of a syndrome. The most common is MEN1, an autosomal dominant disorder resulting from germline pathogenic variants in the MEN1 gene, characterized by involvement of multiple parathyroid glands, pituitary adenomas, and pancreatic tumors (^6^). In MEN1, PHPT generally occurs earlier than in sporadic cases, typically between the second and fourth decades of life (^7-9^). As an autosomal dominant disease, it affects both genders equally (^10^). Parathyroid carcinoma in this syndrome is extremely rare but should always be considered in patients with symptomatic PHPT presenting with the following characteristics: 5- to 10-fold increased serum PTH levels above the reference value, calcium levels above 3.0 mmol/L (12 mg/dL), and large parathyroid lesions on cervical imaging (^11^).

In multiple endocrine neoplasia type 2A (MEN2A), which also demonstrates autosomal dominant inheritance, pathogenic variants are present in the RET gene. MEN2A is characterized by medullary thyroid carcinoma and pheochromocytoma. PHPT may be present in approximately 25% of MEN2A cases and has lower penetrance than in MEN1 (^12^). As in MEN1, onset usually occurs earlier than in the sporadic form, at around 40 years of age, and most cases involve multiple glands. To date, there are no reported cases of parathyroid carcinoma in MEN2A (^13,14^).

Hyperparathyroidism-jaw tumor syndrome arises through autosomal inheritance, and PHPT is the most prevalent feature; the syndrome can also involve ossifying fibromas of the jaw and maxilla and has a higher prevalence of parathyroid carcinoma than MEN1 and MEN2A (approximately 15% of cases) (^15^). In this syndrome, PHPT is typically the first and often the sole symptom, present in nearly all patients, occurring more commonly in late adolescence and young adulthood (^16^), with a mean age at diagnosis of 23 years (^17^). Despite the syndrome’s name, only one third of patients have mandibular or maxillary tumors at diagnosis (^16^).

Familial hypocalciuric hypercalcemia is a rare condition characterized by low urinary calcium levels, with slightly elevated blood calcium and PTH levels. It is caused by pathogenic variants in the CASR gene present in the parathyroid glands and renal tubules. These patients are typically asymptomatic and should be evaluated in the presence of hypocalciuria and a family history of hypercalcemia or parathyroidectomy without criteria for cure (^12^).

Familial isolated hyperparathyroidism is a rare hereditary disorder accounting for approximately 1% of PHPT cases (^18^) and is characterized by PHPT without involvement of other organs, as seen in the more complex hyperparathyroidism syndromes. In our entire cohort, only PHPT was present, with no evidence of other organ or tissue involvement. Initially, FIHP was thought to represent an incomplete form of the known genetically complex syndromes; the concept of this disease has evolved over time (^5^). Currently, FIHP is recognized as a genetically heterogeneous disease, with approximately 30% of kindreds affected by pathogenic variants in MEN1, CDC73, CASR, and, more recently, GCM2 genes (^18^). Hypercalcemia is generally more severe compared to other familial syndromes that are present with PHPT. The onset of hypercalcemia is also earlier than in sporadic cases and may occur at ages similar to those observed in MEN or even during childhood and adolescence (^3^). **Table 4** summarizes the syndromes associated with PHPT and their main characteristics.

**Table 4:** Syndromes associated with primary hyperparathyroidism

	MEN1	MEN2	MEN4	HPT-JT	FHH	FIHP
Inheritance	AD	AD	AD	AD	AD	AD
PHPT prevalence	95%	20%-30%	80%-90%	80%-90%	20% with elevated PTH	100%
Clinical Conditions	Tumors of the parathyroid, pancreatic islet cells and anterior pituitary	Medullary thyroid carcinomas, phaeochromocytomas and parathyroid tumors	Tumors involve the parathyroid glands, anterior pituitary, and gastro-entero-pancreatic neuroendocrine tissues.	Parathyroid adenomas and carcinomas, in association with fibro-osseous jaw tumors, abnormalities; Parathyroid carcinoma in 15% of the cases	Asymptomatic hypercalcemia in association with an inappropriately low urinary calcium excretion	Primary hyperparathyroidism without any association with other endocrinological diseases or tumors

Adapted from Reference 2. MEN: multiple endocrine neoplasia; HPT-JT: hyperparathyroid jaw-tumor syndrome; FHH: familiar hypocalciuric hypercalcemia; FIHP: familial isolated hyperparathyroidism; PHP: primary hyperparathyroidism; AD: autossomic dominant.

The strengths of this study include the elucidation of a rare cause of PHPT through genetic testing and the capacity to conduct genetic counseling, even in asymptomatic cases. A limitation of this study was its retrospective design, which restricted the evaluation of certain patient data and information.

In conclusion, genetic forms of PHPT are rare and may present as part of various syndromes or in isolation. This case series of FIHP highlights a family with six cases of PHPT occurring in younger patients, with recurrence of PHPT in four individuals and one case of parathyroid carcinoma among family members. All patients had a mutation in the CDC73 gene. Proper suspicion and identification of familial PHPT allow for adequate monitoring, clinical guidance, treatment, and genetic counseling for affected kindreds.
